# SOCIAL ISOLATION OF OLDER ADULTS WITH AND WITHOUT PHYSICAL HEALTH CONDITIONS DURING COVID-19

**DOI:** 10.1093/geroni/igad104.2384

**Published:** 2023-12-21

**Authors:** Tina Kilaberia, Yuanyuan Hu, Edward Ratner, Janice Bell

**Affiliations:** New York University, New York City, New York, United States; New York University, New York City, New York, United States; Minneapolis VA Health Care System, Minneapolis, Minnesota, United States; University of California, Davis, Davis, California, United States

## Abstract

Older adults have been disproportionately affected by COVID-19. This study examined health care utilization and social isolation during the pandemic among older adults using written surveys and zoom and phone interviews. We compared two subgroups, those reporting physical health problems (N=12) and those reporting no health problems (N=6). Half of those with physical health problems reported postponing routine or elected healthcare as compared to four (66%) in the subgroup without physical health problems. Seven of 12 (58%) older adults with physical health problems reported isolation specifically from decreased participation in church even though as many lived either in a retirement community or with family, suggesting greater environmental or social supports. This compares to two (33%) in the subgroup without physical health problems reporting isolation from decreased participation in church with only one older adult living in a retirement community. Interviews identified three themes that complement these data: avoiding danger, compound stress (reported only among those with health problems (N=4)), and church as the largest event. These findings suggest that those with physical health problems were less able to delay seeking healthcare, were more likely to feel isolated when unable to attend church services, and suffered from compound stress due to the combination of public health directives and social unrest, such as the George Floyd protests. Religious, faith, or spiritual supports may be important buffers against social isolation during public health emergency, especially for older adults with physical health conditions and when there is concurrent social unrest.

